# Hands-Free Image Capture, Data Tagging and Transfer Using Google Glass: A Pilot Study for Improved Wound Care Management

**DOI:** 10.1371/journal.pone.0121179

**Published:** 2015-04-22

**Authors:** Gabriel Aldaz, Lauren Aquino Shluzas, David Pickham, Ozgur Eris, Joel Sadler, Shantanu Joshi, Larry Leifer

**Affiliations:** 1 Center for Design Research, Stanford University, Stanford, CA, United States of America; 2 Stanford Health Care, Stanford, CA, United States of America; 3 Delft University of Technology, Delft, The Netherlands; University of Pittsburgh, UNITED STATES

## Abstract

Chronic wounds, including pressure ulcers, compromise the health of 6.5 million Americans and pose an annual estimated burden of $25 billion to the U.S. health care system. When treating chronic wounds, clinicians must use meticulous documentation to determine wound severity and to monitor healing progress over time. Yet, current wound documentation practices using digital photography are often cumbersome and labor intensive. The process of transferring photos into Electronic Medical Records (EMRs) requires many steps and can take several days. Newer smartphone and tablet-based solutions, such as Epic Haiku, have reduced EMR upload time. However, issues still exist involving patient positioning, image-capture technique, and patient identification. In this paper, we present the development and assessment of the SnapCap System for chronic wound photography. Through leveraging the sensor capabilities of Google Glass, SnapCap enables hands-free digital image capture, and the tagging and transfer of images to a patient’s EMR. In a pilot study with wound care nurses at Stanford Hospital (n=16), we (i) examined feature preferences for hands-free digital image capture and documentation, and (ii) compared SnapCap to the state of the art in digital wound care photography, the Epic Haiku application. We used the Wilcoxon Signed-ranks test to evaluate differences in mean ranks between preference options. Preferred hands-free navigation features include barcode scanning for patient identification, Z(15) = -3.873, p < 0.001, r = 0.71, and double-blinking to take photographs, Z(13) = -3.606, p < 0.001, r = 0.71. In the comparison between SnapCap and Epic Haiku, the SnapCap System was preferred for sterile image-capture technique, Z(16) = -3.873, p < 0.001, r = 0.68. Responses were divided with respect to image quality and overall ease of use. The study’s results have contributed to the future implementation of new features aimed at enhancing mobile hands-free digital photography for chronic wound care.

## Introduction

Chronic wounds affect 6.5 million Americans and pose a $25 billion annual financial burden to the U.S. health care system [1]. Of particular importance to hospitals are pressure ulcers (bedsores), which along with venous and diabetic ulcers, comprise the vast majority of chronic wounds. A pressure ulcer is “localized injury to the skin and/or underlying tissue usually over a bony prominence, as a result of pressure, or pressure in combination with shear. A number of contributing or confounding factors are also associated with pressure ulcers; the significance of these factors is yet to be elucidated” [2]. Studies have reported that a pressure ulcer extends the length of stay for an acute hospital admission by seven to fifty days [3]. Nearly 60,000 hospital patients in the U.S. die each year from complications due to hospital-acquired pressure ulcers (HAPUs), while the direct treatment costs for HAPUs at an average hospital range between $400,000 and $700,000 [4]. In 2007, the Centers for Medicare and Medicaid Services (CMS) made a policy decision not to pay for the most severe HAPUs [5]. Thus, to reduce the incidence of HAPUs, hospitals are mandating thorough and accurate documentation, which is necessary to determine the degree of wound severity, evaluate the effectiveness of therapies, and modify treatment plans as appropriate.

Increasingly, healthcare facilities are supplementing direct observation with digital photography for assessment and documentation [6]. The National Pressure Ulcer Advisory Panel (NPUAP) strongly encourages that every wound photograph include patient identification, date and time markings, and wound dimensions [7]. In practice, nurses write each patient’s personal identification (ID) information (e.g. name and medical record number (MRN)) on a paper ruler that they hold next to the wound and include in the picture frame.

Nurses commonly use digital cameras for wound photography, but the process is often tedious, time consuming, and not private. At Stanford Hospital, uploading wound images to a patient’s Electronic Medical Record (EMR) typically requires up to two days. Recent advances in mobile technology have significantly reduced the time between taking wound photographs and uploading images to a patient’s EMR. Nonetheless, smartphones and tablets still have a significant drawback for wound photography: they are hands-on devices. Consequently, two to three nurses are required to capture wound images—one to hold a camera, a second to hold a ruler and position the patient, and often a third nurse to assist with patient positioning. Furthermore, the physical handling of cameras increases the likelihood of cross-contamination and patient infections.

**Fig 1:**
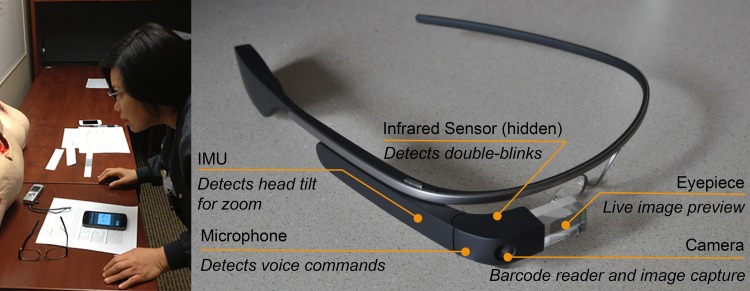
The *SnapCap* System. *SnapCap* is a mobile hands-free application for capturing, tagging, and transferring digital images to a patient’s medical record through leveraging the camera and internal sensors of the Glass wearable computing device (right). A pilot study participant (left) uses both hardware elements of SnapCap, Google Glass and an Android smartphone application, for hands-free wound photography.

The overall aim of this paper is to present the design and lab-based assessment of the SnapCap System, a Google Glass and Android smartphone-based application capable of mobile, hands-free image capture (Fig 1). This system enables nurses to photograph chronic wounds (and other objects of interest), while freeing their hands to position a patient and capture images in a safer, more hygienic manner. Consistent with this philosophy, the SnapCap System also allows speech-to-text annotation for wounds. Since the SnapCap application uses Glass’ integrated camera as a barcode scanner to read a patient’s ID wristband, nurses are no longer required to write patients’ personal identification information on paper rulers. The SnapCap System transmits information encoded in the wristband’s barcode to an EMR to verify patient identity, and tags subsequent wound photographs with the patient’s information. These features reduce critical errors such as image mislabeling or uploading data to the wrong chart.

The main contributions of this paper include:

- The development of a mobile hands-free system that captures, tags, and transfers digital images to a patient’s medical record through leveraging Google Glass’ camera, microphone, inertial measurement unit (IMU), and infrared sensors.
- An examination of user interactions while wearing a head-mounted display and feature preferences (gestural and voice-based) for the purpose of mobile, hands-free digital image capture and documentation.
- A comparison between the SnapCap System and the state of the art in digital wound care photography (Epic Haiku) in a pre-clinical pilot study.

After reviewing related work in this area, we present a detailed description of the technical implementation of SnapCap, including the camera and other relevant sensors. The paper provides an overview of an initial feasibility assessment of the SnapCap System, and a pilot study with wound care nurses at Stanford Hospital. The pilot study demonstrates SnapCap’s usage, uncovers limitations of the current system, and highlights areas for future development.

### Related Work

The SnapCap System presented in this paper builds on prior work in mobile devices for wound photography. The types of devices capable of non-contact wound photography include general-purpose devices (e.g., digital cameras, smartphones, and tablets), wearable (e.g., optical head-mounted displays), and special-purpose devices.

#### General-Purpose Devices

A common way for nurses to take wound photographs is via dedicated digital cameras [8]. After taking photos, a nurse transfers images to a secure, password-protected computer, and deletes the images from the camera. Since digital cameras are not networked, a nurse would then transfer the images via a physical memory card. At Stanford Hospital & Clinics, nurses insert a memory card in a printer to make physical prints. He or she then places each print on a separate piece of paper and fills in relevant information with a pen. Each day, couriers transport a stack of these documents offsite for scanning. It requires up to two days for the images to be uploaded to a patent’s EMR.

Epic Systems (Verona, WI) is a leading provider of EMR software [9]. The Epic Haiku mobile application allows nurses to take photographs using a general-purpose smartphone and immediately upload images to a patient's EMR, where other clinicians may view the images [10]. Photos taken within the Epic Haiku app are not stored on the phone's memory, thereby ensuring compliance with security and privacy protocols [10]. To use Epic Haiku, a nurse typically holds the smartphone in both hands while another nurse holds the paper ruler and positions the patient. Alternatively, one nurse can hold the paper ruler in one hand and the smartphone in the other, in which case pressing the button to take a photograph is often challenging. After taking each image, the nurse must manually enter a text description, which requires one’s full attention and creates a barrier between the nurse and patient. After the patient encounter, the nurse meticulously wipes down all smartphone surfaces to disinfect the device.

MOWA (Mobile Wound Analyzer) is an application for Android smartphones and tablets that takes photos and uses software-based technologies to calculate wound area and identify three types of tissues in the bed of the lesion [11]. Presently, however, MOWA does not integrate with EMRs, thus limiting its utility.

#### Wearable and Special-Purpose Devices

In addition to hand-held digital cameras, a range of studies has demonstrated the use of head-mounted cameras (e.g. GoPro Hero3) for clinical use and education [12]. A major advantage of wearable head-mounted cameras, “is that the user is not restricted in his or her manual and oculomotor actions by camera operation tasks” [13]. This allows the performance of actions in a natural way with increased mobility. Although often WiFi-enabled for remote viewing, wearable cameras typically lack patient identification and EMR integration capabilities for routine clinical care.

Optical head-mounted displays, such as Google Glass (Google, Mountain View, CA), can reflect projected images while allowing the user to see through the device’s eyepiece. Although not widely distributed for consumer use (as of November 2014), a number of proof-of-concept projects and studies using Glass have occurred in the medical field. Medical applications for Glass include remote collaboration between doctors, heads-up viewing of vital signs, medications, and lab reports, as well as live streaming of operations to medical students [14]. Several reports document the live two-way broadcasts of actual patient surgeries by doctors wearing Glass in the operating room [15, 16].

Wearable augmented reality (AR) systems have also been demonstrated in a range of clinical application areas, such as image-guided surgery, pre-operative imaging training, health-related behavior change, and clinical education [17, 18, 19]. Although studies have examined the use of AR systems for both in-patient and remote medical procedures [15, 16, 18], there is a growing need for the development of integrated systems that enable users to seamlessly capture and annotate visual images in a hands-free manner, and integrate this information with a patient’s EMR.

Special-purpose devices usually include sophisticated wound-measurement methods, in addition to photographic capabilities. These devices achieve increased accuracy through vision-based technologies such as stereophotography or structured lighting. For example, MAVIS III (Perry Baromedical, Riviera Beach, FL) is a stereoscopic, hand-held camera that measures the area, volume, and circumference of chronic wounds [20]. Traditionally, special-purpose devices have suffered from one or more of the following drawbacks: they may be expensive, cumbersome to use in a clinical setting, or require significant training time [21].

## Materials and Methods

The methodology for this research included the development of the SnapCap System, with an initial usage/feasibility assessment of the system’s features (conducted in January 2014), a lab-based pilot study with wound care nurses at Stanford Hospital & Clinics (conducted in March 2014), and a follow-up evaluation of speech-to-text for wound annotation (conducted in October 2014).

### Ethics Statement

This research was performed under protocols approved by the Human Subjects Research panel at Stanford University (IRB No. 349 (Panel 2); Protocol ID: 28491). The Institutional Review Board (IRB) approved use of oral consent for this study. The research team provided each study participant with a copy of the research protocol, and verbally asked each participant for his or her consent to participate in the study. Oral consent was manually documented by a member of the research team, prior to the start of each user session. The individuals (photographed) in this manuscript have given written informed consent (as outlined in PLOS consent form) to publish these case details.

### SnapCap System Development

To narrow our research focus, we began with needs finding at Stanford Hospital & Clinics from October to December 2013. We interviewed ten nurses and two physicians, and shadowed four additional nurses (n = 16 clinicians), to generate over 135 needs. We grouped needs into 15 broad clinical areas (shown in Table 1) and ranked each category on a 5-point scale based on degree of importance to the hospital (pain point), alignment with research interests, and feasibility. Within the top-ranking categories, we segmented needs by degree of clinical risk to patients. Our top three needs (in order of low to high patient risk) included: wound and skin care photography, point-of-view sharing during surgery, and vital sign communication during cardiac arrest. We selected chronic wound photography as a target focus area since: (i) a reduction in the incidence of chronic wounds—especially hospital-acquired pressure ulcers—is of paramount concern to healthcare facilities; (ii) chronic wound image capture involves a relatively low degree of clinical risk for patients, thus enabling a solution to be tested and implemented quickly.

**Table 1:** Fifteen clinical areas, in which 135 needs were grouped.

Clinical Area	Degree of importance to the hospital (Pain point)	Alignment with research interests (AR, head-mounted displays)	Feasibility	Mean Score
Documentation (Video Recordings / Photographs)	4	4	5	4.3
Documentation (EMR Data)	5	3	4	4.0
Alerts & Alert Fatigue	5	3	4	4.0
Improving Live Visualization of Cases	3	3	5	3.7
Communication	5	2	4	3.7
Spatial recognition to obtain real-time patient data when near or entering patient rooms	4	3	4	3.7
Training and Education	3	3	4	3.3
Documentation (Decision Support)	4	3	3	3.3
Documentation (Checklists)	3	2	4	3.0
Patient Privacy Issues	5	2	2	3.0
Scheduling and Coordination	2	2	4	2.7
Administering Medications	3	2	3	2.7
Remote Procedures (for emergency responders & home healthcare delivery)	4	2.5	1	2.5
Documentation (Image/Data Overlays)	3	3	1	2.3
Preventing patients from information overload	2	1	1	1.3

The ranking of each clinical area is on a scale of 1–5, with 5 being the highest.

From a development perspective, we were interested in examining the usability features of a head-mounted display and the degree of clinical effectiveness that such a technology affords. We were also interested in examining the efficiency, satisfaction level, benefits and challenges associated with hands-free vs. hands-on image capturing systems. We hypothesized that clinicians could successfully achieve their objective of hands-free digital photography through the use of a head-mounted display, controlled by gestural and voice-based commands; and that such a system could efficiently capture and document wound images, and transmit clinical data to a patient’s EMR.

#### SnapCap System

The SnapCap System includes both a Google Glass application (known as “Glassware”) and an Android smartphone application initially designed to be used by nurses for chronic wound photography [22]. Google Glass [23] was selected as an initial platform for research, since it has an optical head-mounted display that is capable of taking pictures and recording videos using an integrated camera. The device also has the ability to communicate wirelessly via WiFi and Bluetooth. Additional sensors integrated into the device enable the development of augmented reality-based applications. We developed the SnapCap Glassware using the Glass Development Kit (GDK) for Android 4.0.4 (API Level 15, Ice Cream Sandwich), and the smartphone app for Android 4.3 (API Level 18, Jelly Bean).

Working with Google’s new GDK placed constraints on system development. Since interaction via voice commands was limited, we implemented alternative methods to support the hands-free paradigm through gesture-based control. Similarly, working with the camera proved challenging. To preview images before taking them, it was necessary for the system to maintain a continuous video feed to the eyepiece while also allowing for digital zoom, neither of which are standard Glass features. We overcame problems with overheating hardware and an unstable image. Lastly, we had to develop a protocol to transmit image files via Bluetooth, since this was not natively available in the GDK.

**Fig 2:**
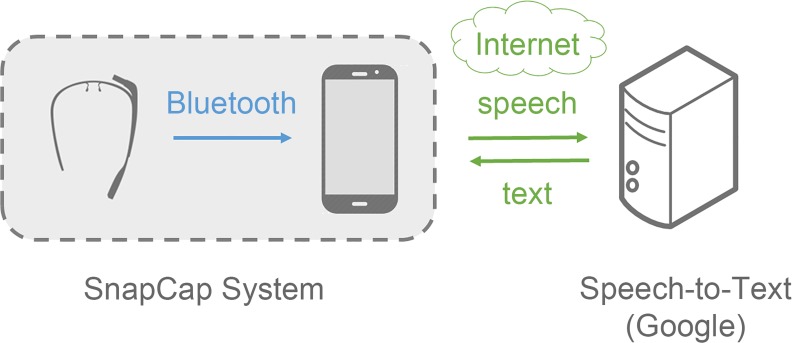
SnapCap prototype system architecture, including Google speech-to-text transcription. The present SnapCap architecture is for prototyping use only. Hospital implementation for routine clinical care would require encrypted communication channels and an interface with proprietary data storage to ensure privacy and security. Ultimately, we intend SnapCap to be platform-agnostic and compatible with a range of EMR systems.

In the current implementation, an Android smartphone serves as a hypothetical EMR that includes a medical database for six fictional patients. The smartphone application communicates with Glass via Bluetooth. Presently, the Bluetooth link is unidirectional, from Glass to the smartphone application, because the app needs to store the images taken with the SnapCap Glassware as well as the associated tags. Speech-to-text conversion takes place on Google servers (Fig 2).

#### SnapCap Smartphone Application

**Fig 3:**
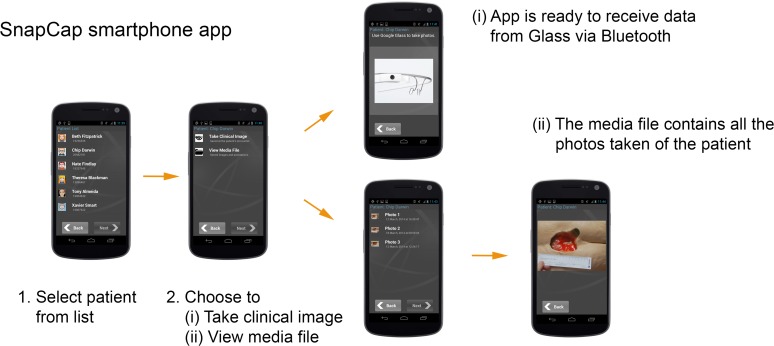
*SnapCap* smartphone application flow. The application is launched prior to each patient encounter.

The SnapCap smartphone application implements relevant features of the EMR, including a patient list, access to a patient database, and image storage in a media file. The password-protected application allows a clinician to select the name of the relevant patient prior to each patient encounter. The clinician can choose to either (i) take a clinical image, whereby the application establishes Bluetooth communication with Google Glass, or (ii) view the patient’s media file. Fig 3 illustrates the application flow.

After choosing to take a clinical image, the nurse is free to place the Android smartphone in his or her pocket and put on Google Glass for the duration of the patient encounter. The photographs taken using Glass, along with the associated tags and voice annotations, immediately transfer via Bluetooth to the smartphone and are stored in the patient’s media file, where they are available for viewing at any time.

#### SnapCap Glassware

The SnapCap Glassware guides a clinician through the steps of sending photographs to the companion smartphone app. The application is launched via the voice command, “*ok glass*, *take clinical image*.” A live camera preview appears in the Glass display, and the application prompts the clinician to look at a barcode encoded with the patient’s MRN. If this MRN matches the MRN of the patient selected by the clinician, the Glass display briefly shows the patient’s name and returns to the live camera preview; otherwise, it displays a warning message. All subsequent images taken are tagged with the patient’s data (e.g. name, MRN, and date of birth).

Once in live camera preview mode, the nurse can zoom in by tilting her head to the right or zoom out by tilting her head to the left. She can take a picture by double-blinking. The picture taken remains on the display for three seconds. Unless the nurse discards the photo (by tilting her head forward), the nurse has the option to add a voice annotation. Lastly, she can tilt her head back to send the image and corresponding data to the hypothetical EMR (the smartphone). In this case, the smartphone app compresses the picture, stores it in the media file, and displays a confirmation message. Otherwise, the nurse can wait three seconds for the live camera preview to return and then take another picture, effectively deleting the previous picture without sending it. The nurse can take as many consecutive pictures as necessary to document the wound.

In summary, the novel features of the SnapCap Glassware include (i) barcode scanning using the Glass camera, and tagging subsequent images with a patient’s personal identification information that is embedded in the barcode, (ii) capturing a live video preview in the Glass eyepiece before a photo is taken, (iii) using a double-blinking gesture to take photographs, utilizing Glass’ IR sensor, and (iv) using a head-tilt gesture (while in the preview mode) to zoom in and out of an image, and a head-tilt gesture to send images to a patient’s EMR, through the use of Glass’ internal measurement unit (IMU) sensor.

#### Google Glass Sensors

Google Glass has four sensors that we have used to implement the SnapCap System for hands-free wound photography: a camera, an inertial measurement unit (IMU), an infrared sensor facing toward the user’s right eye, and a microphone.

*Camera with Digital Zoom*. Albrecht et al. [24] concluded that Glass was efficient for acquiring images, but for photo documentation in forensic medicine, the image quality was inferior to those from a Digital Single-Lens Reflex (DSLR) camera. This finding is perhaps not surprising, considering that Google Glass uses a standard 5-megapixel (2528x1956) smartphone camera with a fixed focal length of 3mm. The Glass camera has a wide-angle lens that captures a field of view too large for most typical medical applications and illustrates the need for zoom [25].

Because Glass has a fixed focus, all zooming is digital and does not lead to a gain in optical resolution. In theory, a photo could be cropped in post-processing to obtain an identical image as one taken after digital zooming, but our preliminary interviews showed that nurses appreciated having the ability to zoom before taking the picture. In practice, we discovered one drawback with zooming before picture taking. Usually, Glass uses burst-mode photography (capturing a rapid sequence of shots to improve dynamic range or reduce noise) when instructed to take a picture [26]. Burst-mode is lost when taking a picture after a zoom, resulting in lower quality photos.

*Barcode reader*. Hospital patients wear ID wristbands printed with a medical record number and corresponding barcode. We implemented a barcode reader based on the open source, cross-platform library ZBar [27]. ZBar works very well with newer smartphones, like the iPhone 5, that have auto-focusing cameras. Since the Google Glass built-in camera has a fixed focus, we used trial and error to find a barcode size that ZBar could read at arm’s length, where the image was focused (we found that objects closer than approximately 30cm would not be in focus). Furthermore, we had to use a flat barcode, rather than the curved barcode on wristbands used at most hospitals.

*Microphone*. The Android 4.0.4 GDK version allows developers to designate a custom voice command to launch an application. SnapCap is launched with the command, “*ok Glass*, *take clinical image*.” We also used the microphone for wound annotation purposes, whereby our app sent audio to the Google servers for speech-to-text conversion. Previous investigators were surprised to find that Glass recognized complex medical terms such as “Microvillous Inclusion Disease” about half the time, but cautioned that for reliable medical use, however, the error rate had to decrease substantially” [25].

*IMU and Infrared Sensor*. Ishimaru et al. [28], who argued that the inertial measurement unit (IMU) and blink detection are the most characteristic features of the Google Glass platform, studied combining head motion with blink frequency for activity recognition. For the purposes of SnapCap, the IMU and infrared sensor each provided a separate input for hands-free interaction. We used the IMU (gyroscope and accelerometer) to measure head tilt and the infrared sensor to detect double blinks.

### Initial Usage and Feasibility Assessment

As an integral part of system development, to better understand how SnapCap might serve the wound documentation needs of nurses, we conducted an initial feasibility and usage assessment of the SnapCap System with five wound care nurses and two physicians at Stanford Hospital (n = 7 participants). Each assessment consisted of two parts, and lasted approximately one hour. Two researchers were present at each session. A digital recording device was used to capture user comments, and researchers took manual notes throughout each session. First, we reviewed the Google Glass navigation basics with each participant. We then asked each participant to complete a series of tasks in order for the research team to qualitatively evaluate the operation of Google Glass, and assess users’ preferences toward new and existing Glass features. Task 1 included the use of voice and touch-based commands to take a picture and record a video, using Glass’s standard features (without image preview and zooming). Task 2 evaluated the use of a customized camera zoom application, with image preview, that allowed users to zoom in and out through blinking one or both eyes in rapid succession. Task 3 assessed the use of the customized camera zoom application with image preview, to zoom in and out through a one-finger gesture (sliding one’s finger back and forth on the Glass touch pad). Task 4 evaluated the use of Glass’ existing historical image retrieval feature, in which users scroll forward on the Glass touchpad to see a list of previously taken images. During each session, the researchers noted which aspects of each task users responded positively or negatively towards, such as actions that were particularly difficult or confusing, actions that users performed with minimal instruction, or actions that appeared exciting to users.

In the second part of the assessment, we asked clinicians to answer six, multi-part questions regarding their preferred methods for capturing, cropping, annotating and retrieving wound images, along with considerations for sterile image-capture techniques. The interview guide (S1 Fig) provides a list of the tasks evaluated and questions asked during each user session (for parts 1 and 2). The research team aggregated user feedback obtained from the seven user sessions, and discussed the user’s task performance (for tasks 1–4) and responses to the multi-part questions.

From this qualitative assessment, we learned that mobile, hands-free operation was critical for wound image capture and annotation. None of the clinicians wanted to touch a head-mounted image-capture system with potentially contaminated gloves. We also learned that, as a baseline functionality requirement, the new system must perform as well as the current Epic Haiku digital image application, which is the state of the art in wound photography at Stanford Hospital. Based on lessons learned from the initial feasibility assessment, we revised the SnapCap Glassware design with modifications and customized features to address users’ needs before conducting the wound care pilot study.

### Image Capture & Documentation

We conducted a two-part within-subjects lab-based pilot study with 16 nurses from Stanford Hospital & Clinics (15 female, 1 male; 11.7 ± 8.7 years of wound care experience). The first part focused on an evaluation of core features of the SnapCap System and a comparison between the use of SnapCap and the iPhone-based Epic Haiku application. The second part of the pilot study involved collecting and analyzing speech-to-text data for wound annotations.

We conducted a lab-based pilot study with hypothetical patients to focus on user interaction preferences for digital image capture and documentation, and to ensure that each nurse captured the same images, as a means of direct comparison between the two applications for wound photography.

Study participants were recruited from a group of wound and ostomy care nurses at Stanford Hospital. None of the nurses had prior Google Glass experience. All sixteen nurses had prior smartphone experience, while three nurses had worked directly with Epic Haiku.

#### Part 1a: SnapCap Feature Evaluation and Application Comparison

**Fig 4:**
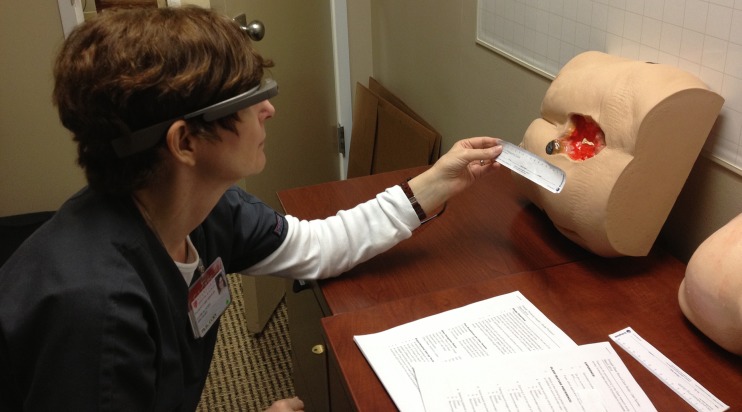
Pilot study participant using SnapCap.

**Fig 5:**
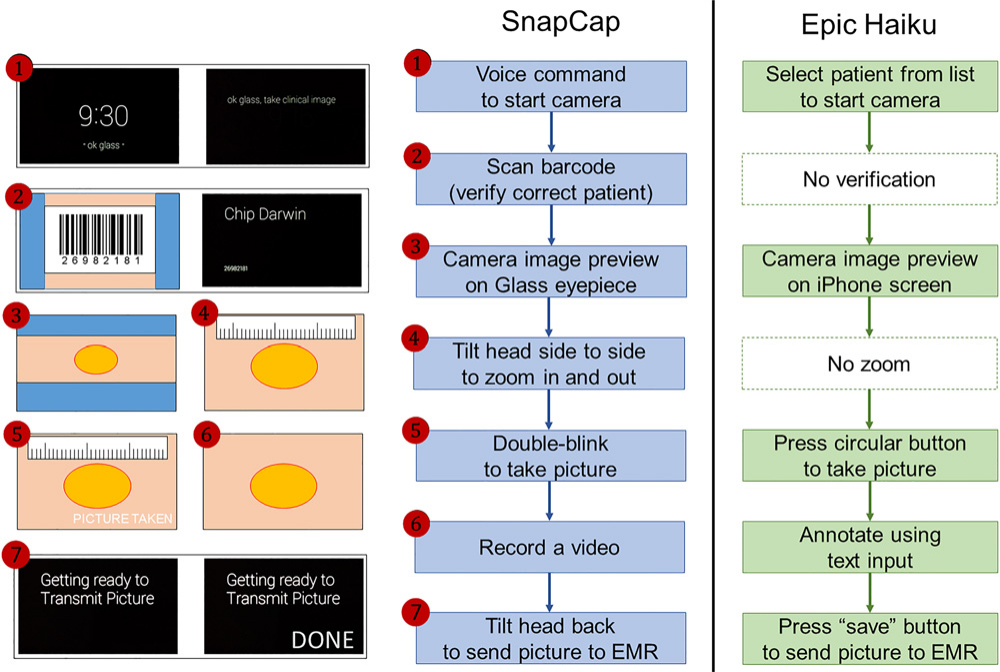
Wound photography flow for SnapCap (left) and Epic Haiku (right).

For part one of the evaluation, we brought each nurse into a hospital room where we had placed two pelvis-only mannequins and an identifying barcode on a table (Fig 4). The mannequins are normally used to train students in the assessment and treatment of pressure ulcer wounds. Two to three researchers were present at each user session. The research team provided each participant with a copy of the study protocol at the start of each session, and asked for each nurse’s consent to participate in the study. The study protocol and user questionnaire (S2 Fig and S3 Fig) are provided as Supporting Information. A researcher explained the purpose of the study, the time breakdown per user session, the Glass navigation basics, and the experimental set-up. A researcher explained the use of both SnapCap and Epic Haiku, and asked each nurse to photograph a wound on a mannequin using (i) SnapCap (running on Glass and a Galaxy Nexus smartphone) and (ii) Epic Haiku (running on an iPhone 4S). The photographs taken by nurses (using SnapCap) were saved in the patient’s hypothetical EMR to examine the quality of each photo taken. For wound annotation, each nurse was asked to use text-based documentation in Epic (per standard documentation practices) and to annotate the wound in a 10-second video recording using the SnapCap system. Fig 5 shows the step-by-step flow in each session. The research team took manual notes and recorded each session using a digital recording device. Photographs and brief videos of nurses using the SnapCap System and Epic Haiku application were also captured. Each user session, consisting of the SnapCap evaluation, application comparison, and a post-task questionnaire, lasted 30–45 minutes per participant, and each subject was given a $15 gift card for his or her study participation.

#### Part 1b: Post-task Questionnaire

Following the use of each system, participants completed a pen-and-paper questionnaire (see S3 Fig) that was segmented into five areas. The questionnaire first asked users to provide their number of years of wound care experience and smart phone experience, and to list mobile applications that they commonly use. The questionnaire then asked participants to share their preferences for (i) current SnapCap system features, (ii) application preferences for SnapCap versus Epic Haiku, and (iii) preferences for the implementation of future SnapCap features. The research team saved the questionnaire data in an Excel spreadsheet for data analysis.

*(i) Glass Feature Preferences*. Specifically, in the first part of the post-task survey, participants were asked to indicate their preference for the following SnapCap features:

1. Barcode scanning for patient identification
2. Voice-based documentation via a brief video recording
3. Double-blinking gesture to take photographs
4. Head-tilt gesture for zooming in and out of an image

Response options were “prefer,” “do not prefer,” or “recommended improvements” (with space to provide improvement recommendations). For each question, a response was required and multiple response options could be selected (e.g., prefer and recommended improvements).

*(ii) Application Preferences*. In the second part of the post-task survey, participants were asked to indicate their preference between SnapCap and Epic Haiku for the following performance dimensions:

1. Considerations for sterile wound image-capture technique
2. Photo-capture capability
3. Image quality
4. Overall Ease of Use

Response options were “Epic Haiku,” “Google Glass,” “no difference,” and “neither.” For each question, a single response was required.

*(iii) Preferences for Future SnapCap Features*. In the third part of the post-task survey, participants were asked to indicate their preferences regarding the perceived benefit of future SnapCap features:

1. The use of a head-mounted display to share and discuss images with colleagues
2. Historical image retrieval for data recall and sharing
3. The use of a dynamic digital ruler inside the Glass eyepiece

Response options were “yes,” “no,” and “no preference.” For each question, a single response was required.

#### Part 2: Speech-to-text Wound Annotation

Following the initial user session, we conducted a 10–15 minute follow-up session at Stanford Hospital with the original sixteen nurse participants, to obtain quantitative data on the performance of Google’s speech-to-text (STT) engine. In these sessions, we asked each nurse to read aloud a wound annotation, consisting of the following two fictitious descriptions, while wearing the Glass head-mounted display:

1. *“This is a stage 3 pressure ulcer, measuring 11 centimeters by 6 centimeters by 3.4 centimeters deep, full-thickness ulceration which probes to bone, circumferential undermining, 2.5 centimeters at 12 o’clock and 0.2 centimeters at 6 o’clock.”*
2. *“Wound bed is approximately 70 percent necrotic tissue, 30 percent non-granulating pale pink wound bed with moderate amount of malodourous brownish red drainage. Wound edges are rolled, closed, non-proliferative. Periwound tissue is intact.”*

Results of the transcription were stored locally in Glass’ built-in memory for analysis.

#### Data Analysis

For the SnapCap feature evaluation and application comparison (Part 1 of the pilot study), we used the Wilcoxon Signed-ranks test, a non-parametric statistical hypothesis test, to evaluate differences in mean ranks between two response options. The analysis was conducted using IBM SPSS statistical software. For the SnapCap feature preference analysis, in which users could select prefer, do not prefer, and/or recommended improvements, we excluded responses in which users only selected “recommended improvements,” since this option did not indicate a specific preference. Instead, the analysis focused on the “prefer” and “do not prefer” options (which may or may not have included recommended improvements) and converted these preferences to ranks for the two possible options. If the “prefer” option was selected, we assigned the rank of 1 to this option, and the rank of 2 to the “do not prefer” option, and vice versa.

Similarly, for an evaluation of the perceived benefit of future SnapCap features, the same analysis method was applied, with the options of yes, no, or no preference. The “no preference” option was excluded, since it did not indicate a specific preference. If “yes” was selected, we assigned the rank of 1 to this option, and the rank of 2 to “no,” and vice versa. For each SnapCap feature preference evaluation (for current and future features), we reported the Wilcoxon p-value and Z-score. For statistically significant results, we reported the effect size using Pearson’s correlation coefficient (r). We plotted nurses’ preferences for various hands-free interaction tasks as the normalized difference between the sum of ranks for feature preferences (based on the SPSS analysis results provided in S4 Fig).

For the application preference analysis, in which users had the choice of Epic Haiku, Google Glass, no difference, or neither, we excluded responses that entailed only the “neither” option because they did not indicate a specific preference. We converted the other three options (Epic Haiku, Google Glass, and no difference) to ranks for the three possible preferences. If the Epic Haiku option was selected, we assigned the rank of 1 to “Epic Haiku” and the rank of 2 to the “Google Glass” option, and vice versa. If the no difference option was selected, we assigned the average rank of 1.5 to both the “Epic Haiku” and the “Google Glass” options. For each application preference comparison, we reported the Wilcoxon p-value, Z-score, and, whenever the results were statistically significant, the effect size using Pearson’s correlation coefficient (r). We graphed nurses’ preferences for SnapCap versus Epic Haiku for wound photography as the normalized difference between the sum of ranks for application preferences (based on the SPSS analysis results provided in S4 Fig).

For both the feature evaluation and application comparison, the difference between sum of ranks is **W** ^**+**^ **−** **W** ^**-**^, where **W** ^**+**^ is the positive sum of ranks ("prefer" and "Google Glass") and **W** ^**-**^ is the negative sum of ranks ("do not prefer" and "Epic Haiku"). The normalized difference between the sum of ranks is (**W** ^**+**^ **−** **W** ^**-**^) / **W**_**max**_, where **W**_**max**_ = n * (n + 1) / 2 = 16 * 17 / 2 = 136.

To examine image quality, we retrieved the images taken by nurses using the SnapCap System and segmented images into the categories: (i) acceptable for clinical use, (ii) blurred, (iii) tilted, (iv) improperly framed, (v) under/over-exposed, and calculated the number and percentage of images in each category.

To examine the qualitative data captured at each user session, the research team reviewed the recommendations for system improvements that were noted by nurses on each questionnaire and added this information to the Excel spreadsheet, by the corresponding system feature. Two team members reviewed the notes and digital recordings captured by researchers at each user session and added comments and observations to the spreadsheet, corresponding to each application’s feature or attribute.

To analyze the Google speech-to-text (STT) data, captured in the follow-up user sessions, we used Word Error Rate (WER) as a standard measure for examining transcription accuracy. WER is defined as the edit distance between the reference word sequence and the sequence emitted by the transcriber. *WER* = (*S* + *D* + *I*) / *N*, where *S*, *D*, and *I* are the number of substitutions, deletions, and insertions, respectively, and *N* is the number of words in the reference [29]. Table 2 illustrates each of these error types.

**Table 2:** Example illustration of the Word Error Rate (WER) analysis, based on the number of deletions, substitutions, and insertions in a transcribed sentence.

**Reference Annotation (REF):**	Thirty	percent	non	granulating	pale	pink	tissue	wound	***	bed
**Actual Transcription (ACT):**	Thirty	percent	***	granulating	pill	pink	tissue	went	to	bed
**Evaluation (EVAL):**			***D***		***S***			***S***	***I***	

An example alignment between the reference annotation (REF) and the actual transcription (ACT), showing deletions (*D*), substitutions (*S*), and insertions (*I*).

(***) are used in the table to denote either a deletion or substitution. For this utterance, there are *N* = 9 words in the reference annotation [WER = (2 + 1 + 1)/9 = 4/9 or 44.4%].

## Results

This section summarizes (i) nurse preferences for hands-free features of the SnapCap System for digital image capture and documentation, and results from the speech-to-text transcription analysis; (ii) a comparison between the SnapCap and the Epic Haiku applications; and (iii) nurse preferences regarding future features for sharing images and data. The results of the SPSS statistical data analysis are provided in supporting documentation (S4 Fig).

### SnapCap System Evaluation: Hands-free Digital Image Capture and Documentation

**Fig 6:**
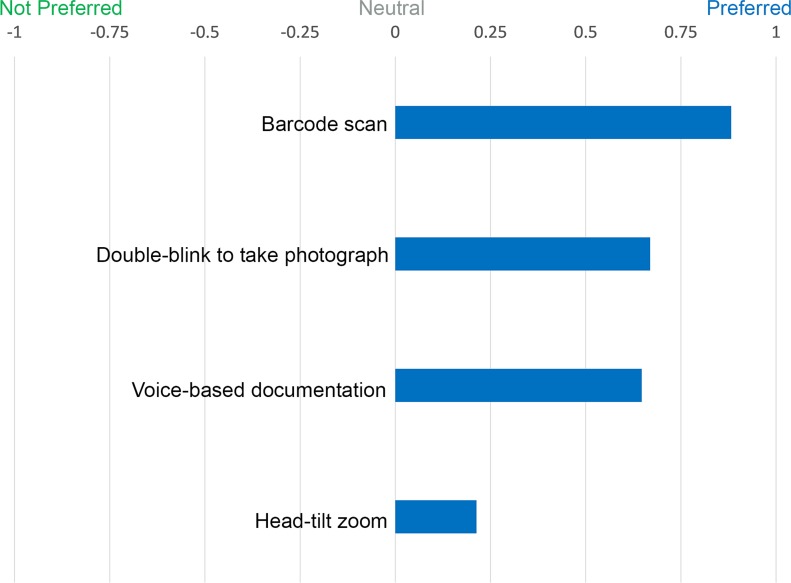
Normalized difference between the sum of ranks for SnapCap feature preferences. Nurses’ preferences for various hands-free interaction tasks as given by the normalized difference between the sum of ranks for feature preferences. A value of +1 indicates that all participants preferred the SnapCap feature, while a value of -1 that all participants did not prefer the SnapCap feature.

We evaluated nurse preferences for features and interactions aimed at enhancing digital image capture and documentation. These included: barcode scanning, voice-based documentation through video, double blinking, and head tilt. Fig 6 illustrates the normalized difference between the sum of ranks for SnapCap feature preferences. In a follow-up study, we examined the accuracy of Google’s STT engine for wound documentation.

#### Barcode Scanning for Patient Identification

A Wilcoxon Signed-ranks test indicated that there was a statistically significant preference for hands-free barcode scanning for routine clinical care, Z(15) = -3.873, p < 0.001, r = 0.71. All sixteen nurses successfully used the SnapCap Glassware to read the patient barcode within four seconds. One nurse indicated that, “Google Glass has the ability to barcode patient identity to reduce errors.”

#### Voice-based Documentation through Video

The data analysis indicated that voice-based documentation through a brief video recording was strongly preferred by nurses, Z(15) = -2.84, p = 0.005, r = 0.52. The qualitative data also indicated that nurses favored the use of voice commands for launching the video recording and image-capture features of the SnapCap System. However, the data revealed that current voice-commands were challenging for participants to use. Only two of sixteen (12.5%) nurses succeeded in using voice commands to launch the video recording feature on their first try. Diverse failure modes included saying an incorrect phrase (3 nurses), saying a phrase too quickly (1 nurse), or saying a phrase too softly (1 nurse). While documenting a wound through a verbally annotated video, nurses frequently commented that the 10-second duration was too short, and that additional time was required.

#### Double Blinking to Take Photographs

Nurses took a photograph (made the camera shutter open and close) by double blinking. Overall, double blinking was well received, with a statistically significant preference for this feature, Z(13) = -3.606, p < 0.001, r = 0.71. One nurse took five or six photographs inadvertently because Google Glass registered her natural eye-blinking rate as double blinking. Another cautioned that double blinking “may be too variable depending on the individual.”

#### Head Tilt for Zooming

Nurses achieved camera zoom in and out by tilting their head to the right and left, respectively. Overall, this was the least preferred hands-free interaction method, Z(10) = -1.897, p = 0.058. One nurse commented that the zoom was slow to respond, and three more indicated that this feature might interfere with their ability to assess wounds or pressure ulcers (for example on the back or the heel), which usually required them to tilt their heads from side to side. They felt that seeing the image zoom in and out on the eyepiece display could potentially interfere with their ability to take a high quality photograph. One nurse had recently been in a car accident and felt slight pain when tilting her head from side to side.

In its present form, the head tilt for zooming interaction suffers from the “Midas Touch” Problem [30]. At first, it seems useful to tilt one’s head from side to side and have the preview zoom in and out. Before long, especially in real-world situations, the constant zooming in and out becomes difficult as users unconsciously move their head to achieve a better view of an object of interest.

#### Speech-to-Text Annotation

When we presented nurses with the possibility of annotating a wound through speech-to-text transcription, they were generally excited about this feature, but anticipated having to access the text for review and editing if necessary.

From data gathered during the follow-up user session, we evaluated the performance of the Google speech-to-text (STT) engine for transcribing wound annotations. Due to technical issues, two of the sixteen annotations had missing data—in one case, the first seventeen words were missing from the transcript; in the other, the last four words were absent. Additionally, we noticed three instances when a nurse uttered an incorrect word, so we removed these individual extraneous words from the data set.

The overall WER of Google’s STT for the fictitious wound annotations was 21.0%. For individual nurses, the WER ranged from 7% to 38% (SD = 10.0%). The word with the highest Individual Word Error Rate, or IWER [31], was “malodorous” (181.3%), which the Google STT reported as “my daughter is” (three instances), “mail order is” (two instances), and “mellow Doris” (one instance). The IWER can be greater than 100% because transcribing “malodorous” to “my daughter is” results in three errors: one substitution and two insertions. “Periwound” had the second highest IWER (140.0%), and Google’s STT had difficulty transcribing the utterance, “periwound tissue,” and generated results such as “Perry won T-shirt” and “Harry born to shoot.” Surprisingly, the word “wound” had the third highest IWER (66.7%), with Google’s STT suggesting “one” instead of “wound” in thirteen instances.

The following subjective comments were worth noting: Five nurses expressed a concern about the performance of the system due to their accents. Three nurses uttered the punctuation—“comma” and “period”—when reading, which the Google STT correctly interpreted. Three nurses thought that the period of silence after which the Google STT considers the utterance complete was too short. One nurse suggested that we load a specialized grammar feature with wound-related terminology to improve speech recognition performance.

From a technical standpoint, the Google STT performed well, in conjunction with the Glass hardware, in a majority of the tests. Fourteen out of sixteen (87.5%) nurses successfully saved the two wound annotations in four attempts or less (although examination of the text files revealed a small amount of missing data on two of the fourteen data files). Two of sixteen nurses (12.5%), however, required more than ten attempts to successfully save the two wound annotations. In both cases, the problem with saving annotations were due to poor network connectivity, which caused the program to hang-up in mid-transcription. The solution required physically moving to another part of the hospital with better wireless reception and recording the annotations in smaller text segments (one sentence at a time) to minimize the chance of malfunction.

### Comparison between SnapCap and Epic Haiku

**Fig 7:**
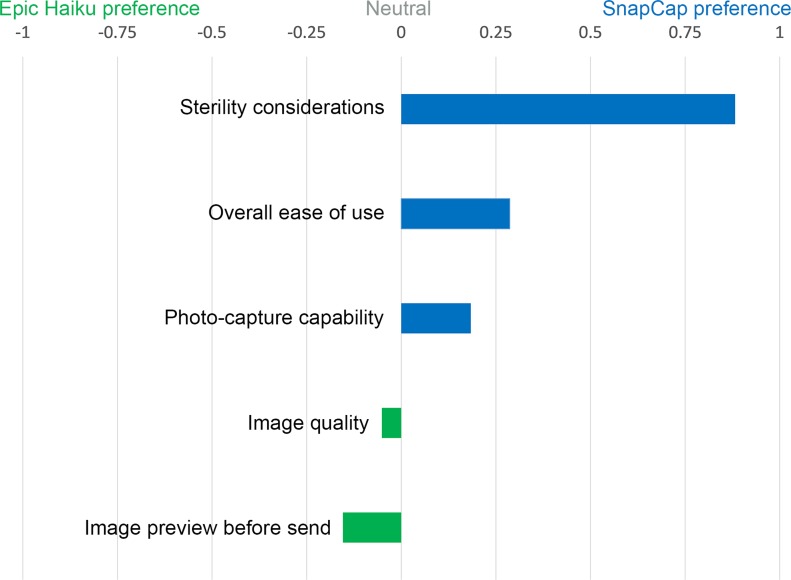
Normalized diference between the sum of ranks for application preferences. Nurses’ preferences when comparing SnapCap and Epic Haiku for wound photography as given by the normalized difference between the sum of ranks for application preferences. A value of +1 indicates that all participants preferred the SnapCap functionality, while a value of -1 that all participants preferred the Epic Haiku functionality.

We compared the SnapCap System and Epic Haiku for digital wound photography. Four aspects we investigated were: (i) sterile image-capture technique, (ii) photo capture capability, (iii) image quality, and (iv) overall ease of use. Fig 7 shows the normalized difference between the sum of ranks for application preferences.

#### Sterile Image-Capture Technique

A Wilcoxon Signed-ranks test indicated that there was a statistically significant preference for the Glass SnapCap System versus Epic Haiku in regard to sterile image-capture technique when photographing wounds, Z(16) = -3.873, p < 0.001, r = 0.68. Comments relating to the SnapCap system included: “Much better experience,” and “Not keen on touching the Glass frame with gloved hands during a consult.”

#### Photo Capture Capability

The results of the data analysis were not statistically significant, and thus insufficient to suggest a difference between the SnapCap System and Epic Haiku for photo capture capability, Z(16) = -1.667, p = 0.096. Also, the data indicated no statistical difference in preferences between the two applications for previewing images after capturing a photograph and before transmitting to an EMR, Z(16) = -0.832, p = 0.405. However, the qualitative data indicated that nurses favored the ability to preview images on a relatively large iPhone screen, as opposed to the smaller Glass display. One nurse commented, “Google Glass has the ability to barcode patient identity to reduce errors, but needs better pixel definition.”

#### Image Quality

**Fig 8:**
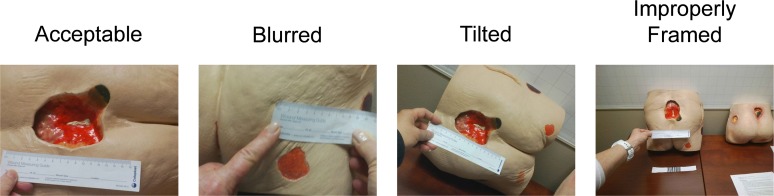
Raw photos taken by nurses with SnapCap exemplifying photo-taking challenges.

Similarly for image quality, the results were not statistically significant, indicating a lack of evidence to support a difference in preferences between the image quality of the SnapCap System and Epic Haiku, Z(15) = -0.816, p = 0.414. Nurses agreed that the quality of photographs taken should be sufficient for making clinical decisions, and qualitatively perceived image quality as a weakness of SnapCap. Using SnapCap, the sixteen nurses transmitted thirty wound photographs from Glass to the smartphone app, of which we considered seventeen of thirty (56.7%) to be of acceptable quality for clinical use. Challenges included blurred (7/30, 23.3%), tilted (3/30, 10%), and improperly framed (3/30, 10%) photos (Fig 8). Blurred shots resulted when the participant took the photo too close to the subject or moved during the shot. The most likely cause for tilted shots was taking a photo while still zooming using head tilt, and improperly framed shots may have been due to inadvertent double-blinking. Interestingly, these challenges are similar to those faced by blind users trying to take photographs when seeking help in obtaining visual information [32]. There were no under/overexposed shots, as the study took place in a hospital room with adequate lighting.

#### Overall Ease of Use

In terms of overall ease of use, the results were not statistically significant; there was insufficient evidence to claim a difference in preferences between the SnapCap system and Epic Haiku for this attribute, Z(14) = -1.732, p = 0.083. Given the lack of familiarity that the nurses had with head-mounted displays, we see the limited difference in ease of use preferences as an encouraging result. One nurse commented that, “Epic Haiku is more ‘normal’ for today's registered nurses, but Google Glass has some great possibilities.” Another nurse called using SnapCap for wound photography, “a much better experience” then Epic Haiku.

### Preferences for Future Image and Data Sharing Features

The survey data captured user preferences regarding potential new features for improved image and data sharing, for digital wound care photography. These include the use of a head-mounted display (HMD) for bi-directional communication with colleagues, features for historical image retrieval for data recall and sharing, and the use of a dynamic digital ruler inside the eyepiece of an HMD.

#### Use of a Head-Mounted Display to Share and Discuss Images

A Wilcoxon Signed-ranks test indicated that there was a statistically significant preference for the future use of head-mounted displays for sharing and discussing wound images among colleagues, in order to obtain real-time feedback on a diagnosis or to aid in clinical decision-making, Z(13) = -3.606, p < 0.001, r = 0.71. One nurse commented that she felt such a system was, “an interesting idea” and another mentioned, “it is a definite possibility.”

#### Historical Image Retrieval for Data Recall and Sharing

The data illustrate a significant preference for historical image retrieval, to achieve time-lapse image recall in a head-mounted display after taking a series of photographs, Z(14) = -3.742, p < 0.001, r = 0.71. Suggested uses for this feature included the ability to see changes in wound margins over time, and to track a wound’s staging and healing progression (for stage 4 to stage 1 pressure ulcers). One nurse commented that it “would show progression or deterioration of the wound.” Another mentioned, “this is very important for staging pressure ulcers—one must know the previous stage so one does not downstage the ulcer.”

#### Dynamic Digital Ruler inside the Glass Eyepiece

The data revealed a significant preference for a digital ruler inside the eyepiece of a head-mounted display, to replace a hand-held paper ruler, Z(13) = -3.051, p = 0.002, r = 0.60. Comments included: “Need to work on accuracy, but yes, that would be great so that you wouldn’t have to hold or dispose of the ruler,” and “Huge help!”

## Discussion

We hypothesized that the SnapCap System, developed using Google Glass, would be an excellent platform for hands-free image capture, data tagging, and transfer for wound care applications. Due to the exploratory nature of this research, we chose to focus on an examination of user preferences as a first step in validating our hypothesis. The performance of the SnapCap System prototype indicates that hands-free photography, for wounds and other applications, is feasible. The data indicate that the nurses who participated in the user study strongly favored SnapCap’s ability to rapidly identify patients through barcode scanning and the use of voice-based commands to launch the application and document wounds.

The overall Word Error Rate for the sample wound annotations was 21.0% and the Google STT correctly recognized every word in the annotations at least once. These results are encouraging, considering the specialized vocabulary of the wound descriptions and the unregulated sound environment in which the sessions took place. However, based on prior research, the WER may be too high for nurses to manually correct the annotations post-recording. In a study of hands-free, speech-based navigation during dictation, in which 20% of the direction-based sequences included a failed command, users reported that the process of manual correction of a dictation was frustrating, labor-intensive, and time-consuming [33]. Similarly, although there were no dictionary errors, the performance of the system for wound annotation would benefit by revising the language model. Incorrect transcription of “periwound tissue” as “Perry won T-shirt” is a homonym error and is always due to a suboptimal language model (a statistical list of possible word adjacencies) [34]. While the word “malodorous” may not appear frequently in annotations, the word “wound” certainly would. A revised language model for wound annotations would assign higher probabilities to the appearance of common wound-related words and phrases.

In general, the positive results obtained in the pilot study underline the need for future work focusing on quantitative metrics, such as time required to perform a task and the success rate in completing a task. Additional areas for future development include: improved voice commands, wound measurement capabilities, contextual time-lapse image recall, and EMR integration.

Since voice commands were a promising interaction method for SnapCap, further investigation in this area is warranted. Specifically, for zooming in and out of wounds, we intend to implement voice commands into the system in lieu of head-tilt gestures. Presently, the GDK is limited to using voice commands to launch applications and converting speech to text at predefined times. As the capabilities of Glass and other AR platforms improve, we expect voice interaction to become a more powerful feature of the SnapCap System.

Although SnapCap’s barcode reader allowed nurses to avoid writing confidential patient information on disposable paper rulers, the current prototype still required nurses to hold a ruler beside a wound to estimate its size. To eliminate the need for paper rulers, we plan to implement a dynamic digital ruler to measure the linear dimensions of wounds or other artifacts of interest. Estimating the area or volume of wounds with greater accuracy, or automatically determining the wound stage, would potentially move SnapCap towards the realm of special-purpose devices such as MAVIS III.

The results of the pilot study also showed that nurses would benefit from a method of contextual time-lapse image recall using the Glass display. In the current prototype, SnapCap transmitted image data to the smartphone but did not receive any data in return. Time-lapse image retrieval would allow nurses to visualize a wound’s staging and healing progression over time.

On a systems level, the SnapCap infrastructure requires the addition of security and privacy features for use in routine clinical care that are specific to each hospital’s EMR system. For this study, we conducted a lab-based evaluation of the SnapCap System to focus on nurse and user interaction preferences for digital image capture and documentation, independent of EMR system-specific functionality. In building on this work, we see the need for a platform-independent, EMR-agnostic system that can be used with both existing and customized head-mounted displays (e.g., Recon Instuments JET, Vuzix M100, SONY Smart Glasses, iOptik). The SnapCap application and data transfer capabilities would be designed to integrate with multiple EMR and clinical decision support systems for seamless interoperability (e.g. Epic, Cerner, McKesson).

In addition to the management of chronic pressure ulcers, we aim to extend the functionality of SnapCap’s interface to meet the needs of image capture during surgery and acute care. Specific applications include point-of-view data sharing during surgery and vital sign communication when patients are suffering from cardiac arrest. Required enhancements include improving the robustness of the head-mounted display to function reliably in a fast-paced hospital setting for an extended period of time, and adding features for use in an operating room’s sterile field and in emergency room settings. Given the image quality limitations of the current Glass-based system, future implementations of SnapCap may require the use of competing head-mounted displays with higher resolution cameras and/or autofocus capability. We also aim to integrate bi-directional communication capabilities into SnapCap to allow users to communicate with colleagues while assessing or diagnosing wounds, in order to enhance real-time clinical decision-making.

## Conclusion

SnapCap enables hands-free digital photography, tagging, speech-to-text image annotation, and the transfer of data to an electronic medical record. In its current implementation, SnapCap leverages Google Glass’ camera and internal sensors to guide clinicians through the process of taking and annotating wound images. This paper documents the SnapCap System architecture and examines user preferences regarding hands-free (voice and gesture-based) interactions for image capture and documentation. In a pilot study with sixteen wound care nurses, we compare the SnapCap Glass application with the state of the art in digital wound care photography.

The data illustrate that nurses strongly favored SnapCap’s ability to rapidly identify patients through barcode scanning. They also favored the use of voice-based commands to launch applications and to document wounds, as well as the double-blinking action to take photographs. However, users expressed mixed views regarding head-tilt gestures for zooming while previewing images. In a head-to-head comparison with the iPhone-based Epic Haiku application, users strongly preferred the SnapCap System for sterile image-capture technique when photographing wounds. Yet, preferences were divided in regard to photo capture capability, image quality, and overall ease of use. The similar ease of use scores for the SnapCap System and Epic Haiku application was promising, given the lack of prior experience that nurses had with head-mounted displays in comparison to smartphones. Based on the study results, this work provides a foundation for the development of new integrated applications for the capture, tagging, and transfer of digital images for wound care and other clinical applications.

## Supporting Information

S1 Fig Interview guide for usability assessment. (PDF)

S2 Fig Study protocol for wound care pilot. (PDF)

S3 Fig User questionnaire for wound care pilot. (PDF)

S4 Fig Data from the SPSS statistical analysis. (PDF)
