# Supplementary material for: Hands-Free Image Capture, Data Tagging and Transfer Using Google Glass: A Pilot Study for Improved Wound Care Management
Source: PLoS One. 2015 Apr 22;10(4):e0121179. doi: 10.1371/journal.pone.0121179 (PMC4406552; doi:10.1371/journal.pone.0121179)
Supplement: S1 Fig — (PDF) [file pone.0121179.s001.pdf]

Thank you for participating in our user study for the SnapCap System, involving Google Glass. This assessment will consist of two parts. The first will involve questions involving the operation of Google Glass for specific tasks. The second will involve a series of questions regarding the use of Glass for wound and skin care management. The overall assessment should take approximately 30 minutes. (A researcher will first wear Glass to demonstrate its features.)

Navigation Basics:

- On Button: press the power button on the inside of the touch pad (small round button).
- Touch Pad: the space with a sensor on the side of the Glass hardware.
- Page selection: use one finger on the touchpad to scroll forward or backwards.
- To select a page one level deeper: tap on the touchpad for the applicable selection.
- To go back: slide your finger from top to bottom on the touch pad.
- If the light on your display turns off, tap once on the side of Glass to start again.
- The button on the right top corner of Glass may be pressed to take a picture instantly.
- When in the home screen, scrolling forward takes you to a photo timeline. Scrolling back, takes you to the Glass settings.

Task 1: Voice and touch commands (photo and video capture without preview and zoom)

1. If required, turn Glass on by pressing the power button on the inside of the touch pad. It is a small round button located towards the end of the touchpad.
2. Please place Google Glass on your head, in the same way that you would wear normal reading glasses.
3. As Glass is starting you will see a screen with "GLASS" written on it.
4. When it is done, you will see a home screen with the current time prominently displayed.
5. Using your left hand, hold a ruler at arm's length in front of you with, and look at the ruler.
6. Say, "Okay Glass, take a picture."
7. Return to the home page. (Slide your right finger from top to bottom on the touch pad to do so.)
8. With your right hand, touch the button on the top right hand corner of Glass to take a second picture.
9. Return to the home page. (Slide your right finger from top to bottom on the touch pad to do so.)
10. Say, "Okay Glass, record a video."

Task 2: Camera Zoom App – Zoom with Blinking

1. To launch the app say: "Ok Glass, Launch Camera Zoom"
2. Using your left hand, hold a ruler at arm's length in front of you with, and look at the ruler with Glass.
3. You will see a camera preview which can be modified to suit your needs (zoom in and zoom out)
4. To zoom in: blink twice in quick succession. You may blink with your right eye only or with both eyes at the same time. The camera will continue to zoom until a maximum level is reached, after which blinking twice will cause the camera to zoom out.
5. Once you have achieved the desired level of zoom, please take a picture by tapping on the touch pad once.

Task 3: Camera Zoom App – Zoom with a Finger Touch

1. While in the Camera Zoom App, please zoom using the touch gesture by scrolling forward on the touch pad with your finger.
2. Zoom out with a touch gesture by scrolling backward on the touch pad, using your finger.
3. Once you have achieved the desired level of zoom, please take a picture by either winking (using your right eye) or by tapping on the touch pad once.

Task 4: Historical Image Retrieval

1. Return to the main screen (slide your finger from top to bottom on the touch pad). You may have to repeat the gesture more than once.
2. Scroll Forwards and you will see a list of previously taken images.

Task 5: Q&A

1. Image Capture Method
    a. What is your preferred image capture method (voice or touch)?

2. Image Cropping Preferences (before or after a photo is taken)
    a. When taking photos, would you prefer to crop an image before taking a photo (through zooming), or manually afterwards – before the image is sent to an EHR?

3. Image Preview

a. Would you prefer to preview an image before taking a photo or to take a photo without previewing the image first (e.g. just taking a photo based on what you see in front of you, not what you see in Glass)?

4. Image Annotation
    a. How do you currently annotate wound and dermatology images?
    b. How do you document a wound's size and location, in relationship to a patient's body?
    c. How could Glass potentially help to improve the annotation process?

5. Historical Image Retrieval
    a. For retrieving patient's images from the EHR, what information would you want to see tagged with each image for fast data retrieval?

6. Sterility
    a. What are some of your concerns regarding the use of image capture technology and wound sterility? What precautions would be needed to maintain a sterile wound care environment when using Glass?

For researcher: To See an Image from Eclipse

Eclipse → Open Perspective → DDMS → File Explorer → mnt → sdcard → GUI
