# Supplementary material for: Hands-Free Image Capture, Data Tagging and Transfer Using Google Glass: A Pilot Study for Improved Wound Care Management
Source: PLoS One. 2015 Apr 22;10(4):e0121179. doi: 10.1371/journal.pone.0121179 (PMC4406552; doi:10.1371/journal.pone.0121179)
Supplement: S2 Fig — (PDF) [file pone.0121179.s002.pdf]

Study Overview

- The purpose of this study is to compare the use of the SnapCap System to Epic Haiku to capture photos for wound and skin care.
- The overall assessment should take approximately 30-45 minutes to complete.
- With your permission, comments will be captured via a digital recording device and a photo of your participation may be captured. If you do not wish to be recorded and/or do not want to be shown in a photo, please let the research team know at any time.
- Your participation is entirely voluntary and you may withdraw from the study at any time.
- A $15 Amazon gift card will be provided for your participation in this study.
- This research has been approved by the Human Subjects Research panel at Stanford University (IRB Protocol I.D. 28491). *No patients will be used in this study.*

Time Breakdown per User Session

5 minutes	Study Overview and Oral Consent
5 minutes	Demonstration of Glass navigation basics
10 minutes	Photo capture & documentation using Glass
10 minutes	Photo capture & documentation using Epic Haiku
5 – 10 minutes	Survey and Incentive form

Glass Navigation Basics

Ok Glass, Take clinical image	Launches photo taking application
Head tilt right (~45 deg)	Zoom in
Head tilt left (~45 deg)	Zoom out
Double blink	Take a picture
Downward swipe	Exit application
Okay Glass, record a video	Captures a 10 second video recording

Experimental Set-up Includes:

- A wound mannequin on a table top in Room H0105J (Nursing Administration)
- 1 pair of Google Glass for the study participant
- A pair of Glass for each researcher for demonstration purposes
- A cell phone with Epic Haiku Playground installed – connected to a hypothetical patient (different from the Glass EHR)
- A digital recording device to capture audio feedback from users
- A copy of the protocol for each participant
- Incentives (gift cards) form for study participants to complete

SnapCap (Google Glass) Image Capture & Documentation Scenario

Researcher 1 will read each step as the participant enacts the scenario.
Researcher 2 (if present) will take notes throughout the study.

Cell phone usage before entering a patient's room:

1. Login to the photo taking application on a cell phone (researchers complete this step with the study participant)
   Username: leave blank
   Password: 4321
   App = electronic health record

2. Before entering a patient's room, please use the cell phone to choose the patient, Chip Darwin, from the list of patients provided.

3. Select 'Take clinical Image' from the smart phone selections.

4. Place the cell phone on the table without logging out of the application.

Photo capture using Google Glass:

5. Put on the Glass head mounted display and approach the hypothetical patient.

6. You should see a clock in the Glass eye piece.

7. Launch the application by quickly saying: Ok Glass, Take clinical image

8. Look at the patient's barcode through the Glass eyepiece (from a distance of 1-2 feet away)

9. Glass will automatically capture the barcode. You should see the patient's name and medical record number (MRN) appear in the eyepiece for a few seconds.

10. The eyepiece will automatically go into a camera preview mode after barcode scanning has occurred.

11. Please place a ruler in front of the wound and view the patient's wound through the Glass eyepiece.

12. To zoom: Tilt your head right to zoom in or left to zoom out. You may hold your head in one position or other to continue zooming.

13. Once you've achieved the desired zoom level, please take a picture by double blinking. The image will freeze in the eyepiece for a second with 'picture taken' visible. Hold a few seconds and tilt your head back about 30 degrees to transmit the image to the hypothetical HER.

14. '<u>Getting ready to transmit picture</u>' should appear in the eyepiece.

    a. Note: In the final application, an opportunity to review images in Glass prior to sending to a patient's health record will be possible.

    b. If no zooming was required prior to taking a photo, a user will need to tilt his or her head upwards by about 30 degrees to transmit the photo to the hypothetical electronic health record.

    c. If zooming was needed, no head tilt is required to transmit photos.

15. As a safeguard to ensure that photos match the correct patient: The smart phone will indicate if it has received the photo, and if the MRN linked with the photo matches the patient that has been previously selected.

16. Once an image has been sent successfully, the words '<u>wound image sent</u>' should appear in the eyepiece and the user is returned to the camera preview mode.

17. If you wish, you may take another photo by zooming and blinking twice.

18. When finished taking photos, please exit the camera application by swiping downward at the side of the Glass headset. (A user will be directed to the Glass home screen)

19. Video documentation. Please record a 10 second video that describes the wound by saying: 'Okay Glass – record a video'

<u>Cell phone use after photos are taken:</u>

1. Look at the cell phone, and ensure that you are on the same screen when you started using Glass.
2. For a historical view of images – Please select 'Media File' on the phone.
3. Open photos one at a time. (time lapse in Glass is planned for a future iteration)

The Glass portion of this test is completed.

Epic Haiku Image Capture & Documentation Scenario:

Researcher 1 will read each step as the participant enacts the scenario.
Researcher 2 (if present) will take notes throughout the study.

1. Please login to the Epic Haiku application on the iphone.
    a. SID:
    b. Password:

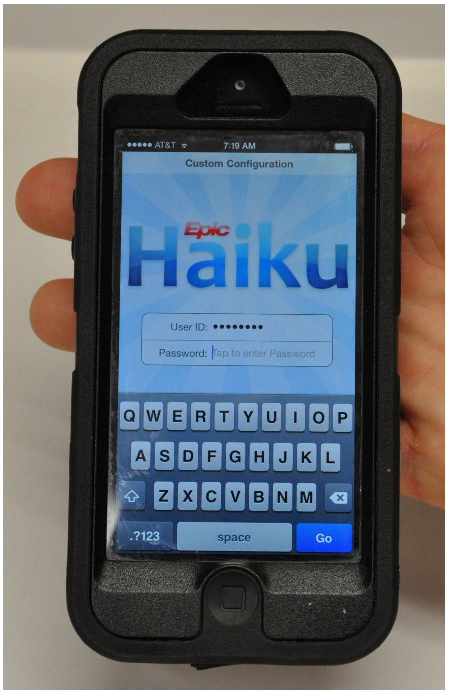

2. Go to: 'My Patient Lists' to access a list of hypothetical patient profiles → Click on any of the profiles shown.

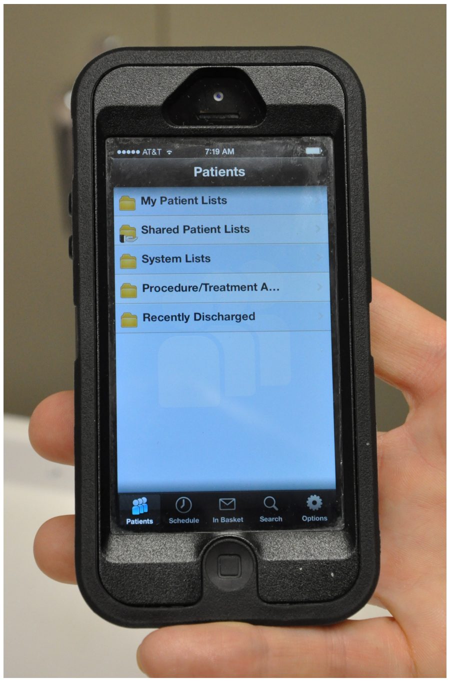

3. Go to Media Capture → Take Clinical Image

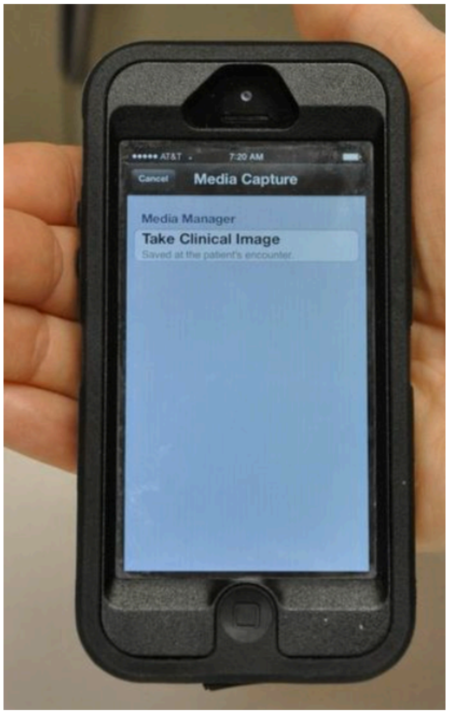

4. Take a photo of a wound on the mannequin

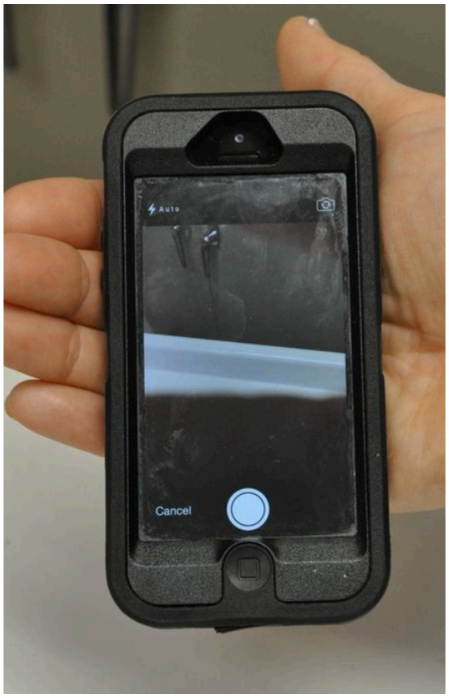

5. Choose to Use (accept) or Retake the photo

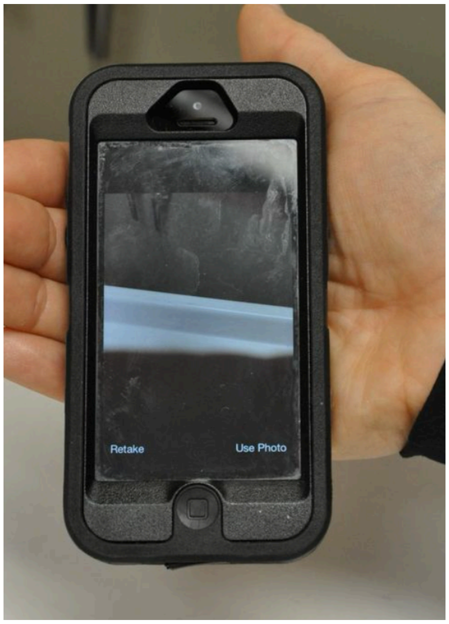

6. See a screen that directs the user to document the wound type

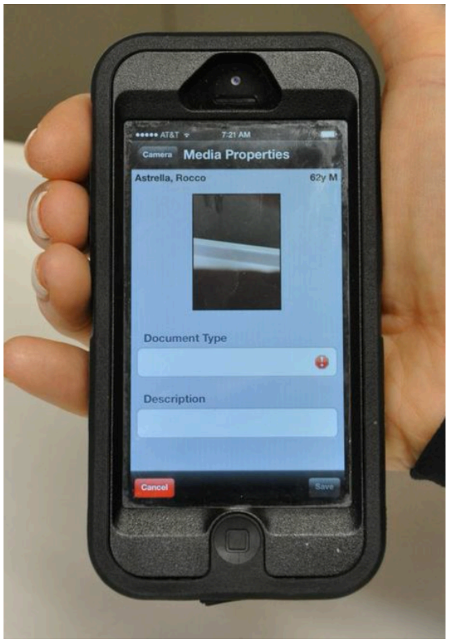

7. Select 'Photo Specimen' to document the wound type from the selection of possible types:

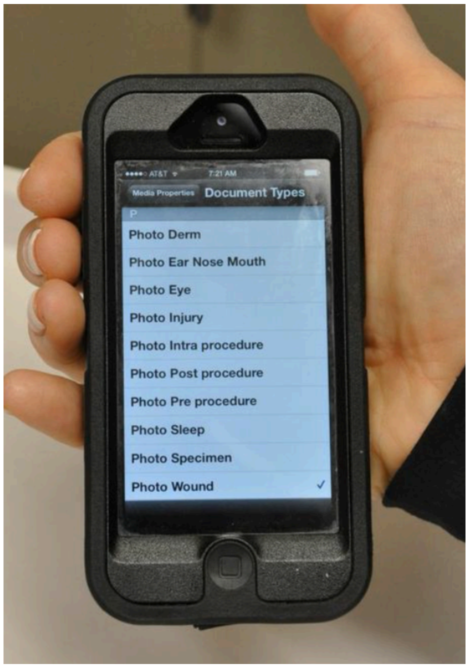

8. Type in a brief text-based description of the wound

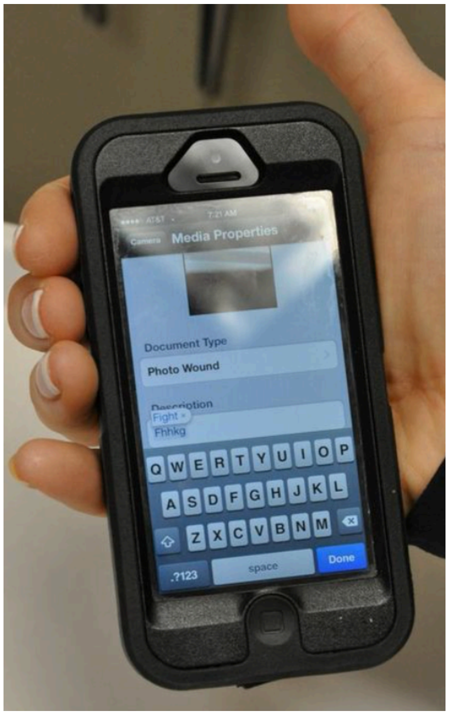

9. Select 'Save' to the send the photo to the hypothetical patient's EHR

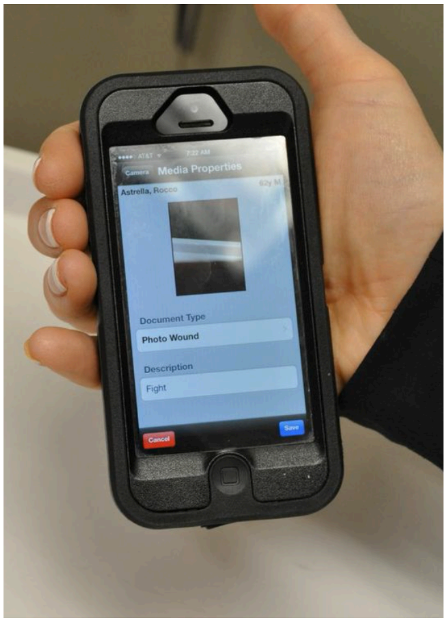

The Epic Haiku portion of this test is completed. Thank you for your participation. Please answer the survey questions provided and complete the incentives form.
