# Supplementary material for: Hands-Free Image Capture, Data Tagging and Transfer Using Google Glass: A Pilot Study for Improved Wound Care Management
Source: PLoS One. 2015 Apr 22;10(4):e0121179. doi: 10.1371/journal.pone.0121179 (PMC4406552; doi:10.1371/journal.pone.0121179)
Supplement: S3 Fig — (PDF) [file pone.0121179.s003.pdf]

SnapCap (Google Glass) Wound Care Study – User Survey
March 2014

Thank you for participating in this research study. Please complete the following survey. It should take about 5-10 minutes to complete.

EXPERIENCE:

1. Estimated number of years of wound care experience:____________________

2. Do you currently use a smart phone application to take wound images (y/n):________

3. If yes, which application do you most commonly use?_____________________

GLASS (SnapCap) FEATURE PREFERENCE:

1. Which of the following photo capture features in Google Glass did you prefer or not prefer for wound and skin care:

   a. Barcode scanning using the head-mounted display
      a. Prefer
      b. Do not prefer
      c. Recommended improvements

   b. Zooming in and out with a head tilt gesture
      a. Prefer
      b. Do not prefer
      c. Recommended improvements

   c. Taking wound images with a double blinking gesture
      a. Prefer
      b. Do not prefer
      c. Recommended improvements

   d. Voice-based documentation through a brief video clip
      a. Prefer
      b. Do not prefer
      c. Recommended improvements

   e. Selecting patients on a smart phone prior to photo taking
      a. Prefer
      b. Do not prefer
      c. Recommended improvements

f. Previewing images on a smart phone after photo capture
    a. Prefer
    b. Do not prefer
    c. Recommended improvements

2. Are there features in the Epic Haiku photo taking application that you would like to have seen in the Glass application?

APPLICATION PREFERENCE:

1. Please select your preferred application for the following metrics:

Overall Ease of Use:
a. Epic Haiku
b. Google Glass
c. Neither
d. No difference

Sterility considerations when handling wounds:
a. Epic Haiku
b. Google Glass
c. Neither
d. No difference

Photo-capture capability:
a. Epic Haiku
b. Google Glass
c. Neither
d. No difference

Ability to preview images before sending to the HER:
e. Epic Haiku
f. Google Glass
g. Neither
h. No difference

Image quality:
i. Epic Haiku
j. Google Glass
k. Neither
l. No difference

PHOTO CAPTURE & DOCUMENTATION PREFERENCE:

1. Please indicate your preferred method of photo taking:
   a. Using gestural commands (e.g. head tilt and blinking)
   b. touch-based commands
   c. voice-activated commands

2. Preferred method of wound documentation:
   a. Voice-based without video (speech to text)
   b. Voice-based with video
   c. Typing (or texting)

3. Do you see a perceived benefit to historical image retrieval for time-lapse image recall, after taking a series of photos over time?
   a. Yes
   b. No
   c. Not sure

4. Would you prefer to see a historical view of images in:
   a. A head mounted display (Glass)
   b. On a smart phone

5. Would you prefer that the ruler be digital (inside the Glass eyepiece):
   a. Yes
   b. No
   c. No preference

6. How do you currently document a wound's size and location, in relationship to a patient's body?

7. Do you foresee potentially using a head-mounted display to share and discuss images among colleagues, in order to obtain real-time feedback on a diagnosis and/or aid in clinical decision-making?
   a. Yes
   b. No
   c. Not sure at this time

EHR INTEGRATION:

1. Do you have recommendations for integrating photo capture via a head-mounted display with a patient's electronic health record?
