# Supplementary material for: Hands-Free Image Capture, Data Tagging and Transfer Using Google Glass: A Pilot Study for Improved Wound Care Management
Source: PLoS One. 2015 Apr 22;10(4):e0121179. doi: 10.1371/journal.pone.0121179 (PMC4406552; doi:10.1371/journal.pone.0121179)
Supplement: S4 Fig — (PDF) [file pone.0121179.s004.pdf]

```
GET
  FILE='D:\oeris\Desktop\GG study 15OCT14.sav'.
DATASET NAME DataSet1 WINDOW=FRONT.
NPAR TESTS
  /WILCOXON=Ease_of_use_EH WITH Ease_of_use_GG (PAIRED)
  /STATISTICS DESCRIPTIVES
  /MISSING ANALYSIS.
```

NPar Tests

[DataSet1] D:\oeris\Desktop\GG study 15OCT14.sav

Descriptive Statistics

	N	Mean	Std. Deviation	Minimum	Maximum
Ease_of_use_EH	14	1.7143	.42582	1.00	2.00
Ease_of_use_GG	14	1.2857	.42582	1.00	2.00

Wilcoxon Signed Ranks Test

Ranks

		N	Mean Rank	Sum of Ranks
Ease_of_use_GG - Ease_of_use_EH	Negative Ranks	9[a]	6.50	58.50
	Positive Ranks	3[b]	6.50	19.50
	Ties	2[c]		
	Total	14		

a. Ease_of_use_GG < Ease_of_use_EH

b. Ease_of_use_GG > Ease_of_use_EH

c. Ease_of_use_GG = Ease_of_use_EH

Test Statistics[a]

	Ease_of_use_GG - Ease_of_use_EH
Z	-1.732[b]
Asymp. Sig. (2-tailed)	.083

a. Wilcoxon Signed Ranks Test

b. Based on positive ranks.

```
NPAR TESTS
  /WILCOXON=Sterility_EH WITH Sterility_GG (PAIRED)
  /STATISTICS DESCRIPTIVES
  /MISSING ANALYSIS.
```

NPar Tests

[DataSet1] D:\oeris\Desktop\GG study 15OCT14.sav

Descriptive Statistics

	N	Mean	Std. Deviation	Minimum	Maximum
Sterility_EH	16	1.9688	.12500	1.50	2.00
Sterility_GG	16	1.0313	.12500	1.00	1.50

Wilcoxon Signed Ranks Test

Ranks

		N	Mean Rank	Sum of Ranks
Sterility_GG - Sterility_EH	Negative Ranks	15[a]	8.00	120.00
	Positive Ranks	0[b]	.00	.00
	Ties	1[c]		
	Total	16		

a. Sterility_GG < Sterility_EH

b. Sterility_GG > Sterility_EH

c. Sterility_GG = Sterility_EH

Test Statistics[a]

	Sterility_GG - Sterility_EH
Z	-3.873[b]
Asymp. Sig. (2-tailed)	.000

a. Wilcoxon Signed Ranks Test

b. Based on positive ranks.

```
NPAR TESTS
  /WILCOXON=PhotoCapture_EH WITH PhotoCapture_GG (PAIRED)
  /STATISTICS DESCRIPTIVES
  /MISSING ANALYSIS.
```

NPar Tests

```
[DataSet1] D:\oeris\Desktop\GG study 15OCT14.sav
```

Descriptive Statistics

	N	Mean	Std. Deviation	Minimum	Maximum
PhotoCapture_EH	16	1.6563	.35208	1.00	2.00
PhotoCapture_GG	16	1.3437	.35208	1.00	2.00

Wilcoxon Signed Ranks Test

Ranks

		N	Mean Rank	Sum of Ranks
PhotoCapture_GG - PhotoCapture_EH	Negative Ranks	7[a]	5.00	35.00
	Positive Ranks	2[b]	5.00	10.00
	Ties	7[c]		
	Total	16		

a. PhotoCapture_GG < PhotoCapture_EH

b. PhotoCapture_GG > PhotoCapture_EH

c. PhotoCapture_GG = PhotoCapture_EH

Test Statistics[a]

	PhotoCapture _GG - PhotoCapture _EH
Z	-1.667[b]
Asymp. Sig. (2-tailed)	.096

a. Wilcoxon Signed Ranks Test

b. Based on positive ranks.

```
NPAR TESTS
  /WILCOXON=Preview_EH WITH Preview_GG (PAIRED)
  /STATISTICS DESCRIPTIVES
  /MISSING ANALYSIS.
```

NPar Tests

```
[DataSet1] D:\oeris\Desktop\GG study 15OCT14.sav
```

Descriptive Statistics

	N	Mean	Std. Deviation	Minimum	Maximum
Preview_EH	16	1.4063	.45529	1.00	2.00
Preview_GG	16	1.5938	.45529	1.00	2.00

Wilcoxon Signed Ranks Test

Ranks

		N	Mean Rank	Sum of Ranks
Preview_GG - Preview_EH	Negative Ranks	5[a]	7.00	35.00
	Positive Ranks	8[b]	7.00	56.00
	Ties	3[c]		
	Total	16		

a. Preview_GG < Preview_EH

b. Preview_GG > Preview_EH

c. Preview_GG = Preview_EH

Test Statistics[a]

	Preview_GG - Preview_EH
Z	-.832[b]
Asymp. Sig. (2-tailed)	.405

a. Wilcoxon Signed Ranks Test

b. Based on negative ranks.

```
NPAR TESTS
  /WILCOXON=Image_EH WITH Image_GG (PAIRED)
  /STATISTICS DESCRIPTIVES
  /MISSING ANALYSIS.
```

NPar Tests

```
[DataSet1] D:\oeris\Desktop\GG study 15OCT14.sav
```

Descriptive Statistics

	N	Mean	Std. Deviation	Minimum	Maximum
Image_EH	15	1.4333	.31997	1.00	2.00
Image_GG	15	1.5667	.31997	1.00	2.00

Wilcoxon Signed Ranks Test

Ranks

		N	Mean Rank	Sum of Ranks
Image_GG - Image_EH	Negative Ranks	2[a]	3.50	7.00
	Positive Ranks	4[b]	3.50	14.00
	Ties	9[c]		
	Total	15		

a. Image_GG < Image_EH

b. Image_GG > Image_EH

c. Image_GG = Image_EH

Test Statistics[a]

	Image_GG - Image_EH
Z	-.816[b]
Asymp. Sig. (2-tailed)	.414

a. Wilcoxon Signed Ranks Test

b. Based on negative ranks.

```
NPAR TESTS
  /WILCOXON=Benefit_over_historical_YES WITH Benefit_over_historical_NO (P
AIRED)
```

```
  /STATISTICS DESCRIPTIVES
  /MISSING ANALYSIS.
```

NPar Tests

```
[DataSet1] D:\oeris\Desktop\GG study 15OCT14.sav
```

Descriptive Statistics

	N	Mean	Std. Deviation	Minimum	Maximum
Benefit_over_historical_YES	14	1.0000	.00000	1.00	1.00
Benefit_over_historical_NO	14	2.0000	.00000	2.00	2.00

Wilcoxon Signed Ranks Test

Ranks

		N	Mean Rank	Sum of Ranks
Benefit_over_historical_NO - Benefit_over_historical_YES	Negative Ranks	0[a]	.00	.00
	Positive Ranks	14[b]	7.50	105.00
	Ties	0[c]		
	Total	14		

a. Benefit_over_historical_NO < Benefit_over_historical_YES

b. Benefit_over_historical_NO > Benefit_over_historical_YES

c. Benefit_over_historical_NO = Benefit_over_historical_YES

Test Statistics[a]

	Benefit_over_historical_NO - Benefit_over_historical_YES
Z	-3.742[b]
Asymp. Sig. (2-tailed)	.000

a. Wilcoxon Signed Ranks Test

b. Based on negative ranks.

```
NPAR TESTS
  /WILCOXON=Preferred_Display_headmounted WITH Preferred_Display_smartphon
e (PAIRED)
  /STATISTICS DESCRIPTIVES
  /MISSING ANALYSIS.
```

NPar Tests

```
[DataSet1] D:\oeris\Desktop\GG study 15OCT14.sav
```

Descriptive Statistics

	N	Mean	Std. Deviation	Minimum	Maximum
Preferred_Display_headmounted	15	1.6667	.48795	1.00	2.00
Preferred_Display_smartphone	15	1.3333	.48795	1.00	2.00

Wilcoxon Signed Ranks Test

Ranks

		N	Mean Rank	Sum of Ranks
Preferred_Display_smartphone - Preferred_Display_headmounted	Negative Ranks	10[a]	8.00	80.00
	Positive Ranks	5[b]	8.00	40.00
	Ties	0[c]		
	Total	15		

a. Preferred_Display_smartphone < Preferred_Display_headmounted

b. Preferred_Display_smartphone > Preferred_Display_headmounted

c. Preferred_Display_smartphone = Preferred_Display_headmounted

Test Statistics[a]

	Preferred_Display_smartphone - Preferred_Display_headmounted
Z	-1.291[b]
Asymp. Sig. (2-tailed)	.197

a. Wilcoxon Signed Ranks Test

b. Based on positive ranks.

```
NPAR TESTS
  /WILCOXON=Digital_Ruler_YES WITH Digital_Ruler_NO (PAIRED)
  /STATISTICS DESCRIPTIVES
  /MISSING ANALYSIS.
```

NPar Tests

```
[DataSet1] D:\oeris\Desktop\GG study 15OCT14.sav
```

Descriptive Statistics

	N	Mean	Std. Deviation	Minimum	Maximum
Digital_Ruler_YES	13	1.0769	.27735	1.00	2.00
Digital_Ruler_NO	13	1.9231	.27735	1.00	2.00

Wilcoxon Signed Ranks Test

Ranks

		N	Mean Rank	Sum of Ranks
Digital_Ruler_NO - Digital_Ruler_YES	Negative Ranks	1[a]	7.00	7.00
	Positive Ranks	12[b]	7.00	84.00
	Ties	0[c]		
	Total	13		

a. Digital_Ruler_NO < Digital_Ruler_YES

b. Digital_Ruler_NO > Digital_Ruler_YES

c. Digital_Ruler_NO = Digital_Ruler_YES

Test Statistics[a]

	Digital_Ruler_NO - Digital_Ruler_YES
Z	-3.051[b]
Asymp. Sig. (2-tailed)	.002

a. Wilcoxon Signed Ranks Test

b. Based on negative ranks.

```
NPAR TESTS
  /WILCOXON=Future_HMD_use_YES WITH Future_HMD_use_NO (PAIRED)
  /STATISTICS DESCRIPTIVES
  /MISSING ANALYSIS.
```

NPar Tests

```
[DataSet1] D:\oeris\Desktop\GG study 15OCT14.sav
```

Descriptive Statistics

	N	Mean	Std. Deviation	Minimum	Maximum
Future_HMD_use_YES	13	1.0000	.00000	1.00	1.00
Future_HMD_use_NO	13	2.0000	.00000	2.00	2.00

Wilcoxon Signed Ranks Test

Ranks

		N	Mean Rank	Sum of Ranks
Future_HMD_use_NO - Future_HMD_use_YES	Negative Ranks	0[a]	.00	.00
	Positive Ranks	13[b]	7.00	91.00
	Ties	0[c]		
	Total	13		

a. Future_HMD_use_NO < Future_HMD_use_YES

b. Future_HMD_use_NO > Future_HMD_use_YES

c. Future_HMD_use_NO = Future_HMD_use_YES

Test Statistics[a]

	Future_HMD_use_NO - Future_HMD_use_YES
Z	-3.606[b]
Asymp. Sig. (2-tailed)	.000

a. Wilcoxon Signed Ranks Test

b. Based on negative ranks.

```
NPAR TESTS
  /WILCOXON=Barcode_Scanning_LIKE WITH Barcode_Scanning_DISLIKE (PAIRED)
  /STATISTICS DESCRIPTIVES
  /MISSING ANALYSIS.
```

NPar Tests

```
[DataSet1] D:\oeris\Desktop\GG study 15OCT14.sav
```

Descriptive Statistics

	N	Mean	Std. Deviation	Minimum	Maximum
Barcode_Scanning_LIKE	15	1.0000	.00000	1.00	1.00
Barcode_Scanning_DISLIKE	15	2.0000	.00000	2.00	2.00

Wilcoxon Signed Ranks Test

Ranks

		N	Mean Rank	Sum of Ranks
Barcode_Scanning_DISLIKE - Barcode_Scanning_LIKE	Negative Ranks	0[a]	.00	.00
	Positive Ranks	15[b]	8.00	120.00
	Ties	0[c]		
	Total	15		

a. Barcode_Scanning_DISLIKE < Barcode_Scanning_LIKE

b. Barcode_Scanning_DISLIKE > Barcode_Scanning_LIKE

c. Barcode_Scanning_DISLIKE = Barcode_Scanning_LIKE

Test Statistics[a]

	Barcode_Scanning_DISLIKE - Barcode_Scanning_LIKE
Z	-3.873[b]
Asymp. Sig. (2-tailed)	.000

a. Wilcoxon Signed Ranks Test

b. Based on negative ranks.

```
NPAR TESTS
  /WILCOXON=Zooming_LIKE WITH Zooming_DISLIKE (PAIRED)
  /STATISTICS DESCRIPTIVES
  /MISSING ANALYSIS.
```

NPar Tests

```
[DataSet1] D:\oeris\Desktop\GG study 15OCT14.sav
```

Descriptive Statistics

	N	Mean	Std. Deviation	Minimum	Maximum
Zooming_LIKE	10	1.2000	.42164	1.00	2.00
Zooming_DISLIKE	10	1.8000	.42164	1.00	2.00

Wilcoxon Signed Ranks Test

Ranks

		N	Mean Rank	Sum of Ranks
Zooming_DISLIKE - Zooming_LIKE	Negative Ranks	2[a]	5.50	11.00
	Positive Ranks	8[b]	5.50	44.00
	Ties	0[c]		
	Total	10		

a. Zooming_DISLIKE < Zooming_LIKE

b. Zooming_DISLIKE > Zooming_LIKE

c. Zooming_DISLIKE = Zooming_LIKE

Test Statistics[a]

	Zooming_DISLIKE - Zooming_LIKE
Z	-1.897[b]
Asymp. Sig. (2-tailed)	.058

a. Wilcoxon Signed Ranks Test

b. Based on negative ranks.

```
NPAR TESTS
  /WILCOXON=DoubleBlink_LIKE WITH DoubleBlink_DISLIKE (PAIRED)
  /STATISTICS DESCRIPTIVES
  /MISSING ANALYSIS.
```

NPar Tests

```
[DataSet1] D:\oeris\Desktop\GG study 15OCT14.sav
```

Descriptive Statistics

	N	Mean	Std. Deviation	Minimum	Maximum
DoubleBlink_LIKE	13	1.0000	.00000	1.00	1.00
DoubleBlink_DISLIKE	13	2.0000	.00000	2.00	2.00

Wilcoxon Signed Ranks Test

Ranks

		N	Mean Rank	Sum of Ranks
DoubleBlink_DISLIKE - DoubleBlink_LIKE	Negative Ranks	0[a]	.00	.00
	Positive Ranks	13[b]	7.00	91.00
	Ties	0[c]		
	Total	13		

a. DoubleBlink_DISLIKE < DoubleBlink_LIKE

b. DoubleBlink_DISLIKE > DoubleBlink_LIKE

c. DoubleBlink_DISLIKE = DoubleBlink_LIKE

Test Statistics[a]

	DoubleBlink_DISLIKE - DoubleBlink_LIKE
Z	-3.606[b]
Asymp. Sig. (2-tailed)	.000

a. Wilcoxon Signed Ranks Test

b. Based on negative ranks.

```
NPAR TESTS
  /WILCOXON=VoiceDocumentation_LIKE WITH VoiceDocumentation_DISLIKE (PAIRE
D)
  /STATISTICS DESCRIPTIVES
  /MISSING ANALYSIS.
```

NPar Tests

```
[DataSet1] D:\oeris\Desktop\GG study 15OCT14.sav
```

Descriptive Statistics

	N	Mean	Std. Deviation	Minimum	Maximum
VoiceDocumentation_LIKE	15	1.1333	.35187	1.00	2.00
VoiceDocumentation_DISLIKE	15	1.8667	.35187	1.00	2.00

Wilcoxon Signed Ranks Test

Ranks

		N	Mean Rank	Sum of Ranks
VoiceDocumentation_DISLIKE - VoiceDocumentation_LIKE	Negative Ranks	2[a]	8.00	16.00
	Positive Ranks	13[b]	8.00	104.00
	Ties	0[c]		
	Total	15		

a. VoiceDocumentation_DISLIKE < VoiceDocumentation_LIKE

b. VoiceDocumentation_DISLIKE > VoiceDocumentation_LIKE

c. VoiceDocumentation_DISLIKE = VoiceDocumentation_LIKE

Test Statistics[a]

	VoiceDocumentation_DISLIKE - VoiceDocumentation_LIKE
Z	-2.840[b]
Asymp. Sig. (2-tailed)	.005

a. Wilcoxon Signed Ranks Test

b. Based on negative ranks.

```
NPAR TESTS
  /WILCOXON=Smartphone_Patient_Select_LIKE WITH Smartphone_Patient_Select_
DISLIKE (PAIRED)
  /STATISTICS DESCRIPTIVES
  /MISSING ANALYSIS.
```

NPar Tests

```
[DataSet1] D:\oeris\Desktop\GG study 15OCT14.sav
```

Descriptive Statistics

	N	Mean	Std. Deviation	Minimum	Maximum
Smartphone_Patient_Select_LIKE	15	1.0667	.25820	1.00	2.00
Smartphone_Patient_Select_DISLIKE	15	1.9333	.25820	1.00	2.00

Wilcoxon Signed Ranks Test

Ranks

		N	Mean Rank	Sum of Ranks
Smartphone_Patient_Select_DISLIKE - Smartphone_Patient_Select_LIKE	Negative Ranks	1[a]	8.00	8.00
	Positive Ranks	14[b]	8.00	112.00
	Ties	0[c]		
	Total	15		

a. Smartphone_Patient_Select_DISLIKE < Smartphone_Patient_Select_LIKE

b. Smartphone_Patient_Select_DISLIKE > Smartphone_Patient_Select_LIKE

c. Smartphone_Patient_Select_DISLIKE = Smartphone_Patient_Select_LIKE

Test Statistics[a]

	Smartphone_Patient_Select_DISLIKE - Smartphone_Patient_Select_LIKE
Z	-3.357[b]
Asymp. Sig. (2-tailed)	.001

a. Wilcoxon Signed Ranks Test

b. Based on negative ranks.

```
NPAR TESTS
  /WILCOXON=Smartphone_Preview_LIKE WITH Smartphone_Preview_DISLIKE (PAIRE
D)
  /STATISTICS DESCRIPTIVES
  /MISSING ANALYSIS.
```

NPar Tests

```
[DataSet1] D:\oeris\Desktop\GG study 15OCT14.sav
```

Descriptive Statistics

	N	Mean	Std. Deviation	Minimum	Maximum
Smartphone_Preview_LIKE	14	1.0714	.26726	1.00	2.00
Smartphone_Preview_DISLIKE	14	1.9286	.26726	1.00	2.00

Wilcoxon Signed Ranks Test

Ranks

		N	Mean Rank	Sum of Ranks
Smartphone_Preview_DISLIKE - Smartphone_Preview_LIKE	Negative Ranks	1[a]	7.50	7.50
	Positive Ranks	13[b]	7.50	97.50
	Ties	0[c]		
	Total	14		

a. Smartphone_Preview_DISLIKE < Smartphone_Preview_LIKE

b. Smartphone_Preview_DISLIKE > Smartphone_Preview_LIKE

c. Smartphone_Preview_DISLIKE = Smartphone_Preview_LIKE

Test Statistics[a]

	Smartphone_Preview_DISLIKE - Smartphone_Preview_LIKE
Z	-3.207[b]
Asymp. Sig. (2-tailed)	.001

a. Wilcoxon Signed Ranks Test

b. Based on negative ranks.
